# The Concurrence of Typhoid and Malarial Fever

**Published:** 1900-09-15

**Authors:** 


### THE CONCURRENCE OF TYPHOID AND MALARIAL FEVER.

In view of the statement made in Osler's "Principles and Practice of Medicine" that, among a large number of cases of typhoid fever in which blood examinations were made, in not a single instance were the plasmodia of malaria found, although many of the patients came from malarious districts, it is well to note that Major M. T. Yarr,[1] R.A.M.C., records a case of apparently mild but well-marked typhoid fever, in which the malaria parasite was discovered. From about the twenty-first day his blood was examined, and a few crescents were seen, but very few. About a week later, however, he had some rigors followed by shivering, and then numerous pigmented parasites of the kind figured by Marchiafava and Bignami as "pigmented malignant quotidian" were found.

[1] Brit. Med. Jour., Sept. 8.

